# Mitochondrial Ca^2+^ Signaling and Bioenergetics in Alzheimer’s Disease

**DOI:** 10.3390/biomedicines10123025

**Published:** 2022-11-24

**Authors:** Nikita Arnst, Nelly Redolfi, Annamaria Lia, Martina Bedetta, Elisa Greotti, Paola Pizzo

**Affiliations:** 1 Department of Biomedical Sciences, University of Padova, 35131 Padua, Italy; 2 Neuroscience Institute, Italian National Research Council (CNR), 35131 Padua, Italy; 3 Padova Neuroscience Center (PNC), University of Padova, 35131 Padua, Italy; 4 Study Centre for Neurodegeneration (CESNE), University of Padova, 35131 Padua, Italy

**Keywords:** calcium, Alzheimer’s disease, mitochondria, bioenergetics, neuron, microglia, astrocyte, AD mouse model, iPSCs

## Abstract

Alzheimer’s disease (AD) is a hereditary and sporadic neurodegenerative illness defined by the gradual and cumulative loss of neurons in specific brain areas. The processes that cause AD are still under investigation and there are no available therapies to halt it. Current progress puts at the forefront the “calcium (Ca^2+^) hypothesis” as a key AD pathogenic pathway, impacting neuronal, astrocyte and microglial function. In this review, we focused on mitochondrial Ca^2+^ alterations in AD, their causes and bioenergetic consequences in neuronal and glial cells, summarizing the possible mechanisms linking detrimental mitochondrial Ca^2+^ signals to neuronal death in different experimental AD models.

## 1. Introduction

Mitochondria are well-known organelles that function as the powerhouse of the cell. They occupy approximately 20% of the cell volume, although their number and size vary from cell to cell. Their presence is massive in excitable cells, which rely strongly on oxidative phosphorylation (OXPHOS) to produce the adenosine triphosphate (ATP) necessary to accomplish their physiological functions [1]. However, mitochondria are much more than mere producers of energy. Decades of research have shown that mitochondria represent a formidable intracellular signaling platform that orchestrates various cellular functions. They are crucial, for example, in calcium (Ca^2+^) handling, regulation of cell response to stress, autophagy and cell death. In these processes, their tight interaction with other organelles, in particular the endoplasmic reticulum (ER), is fundamental [2,3]. The central role of mitochondria in cellular physiology is such that alterations in their functionality are involved in the pathogenesis of various disorders, including Alzheimer’s disease (AD).

AD is the most common neurodegenerative disease caused by poorly known pathogenic mechanisms, affecting around 10% of the population aged more than 65 in the United States [4] and around 5% of the European population [5]. Although the sporadic form (SAD) is the most prevalent, the presence of mutations in the amyloid precursor protein (*APP*), presenilin-1 (*PSEN1*) and presenilin-2 (*PSEN2*) genes are marks of familial cases (FAD). FAD forms represent a small percentage (2–3%) of AD cases, and *PSEN1* and *PSEN2* mutations account for more than 80% of genetic lesions [6]. FAD is characterized by an early onset and a worse prognosis compared to SAD, despite that they share many clinical manifestations and pathological hallmarks, i.e., accumulation and deposition of brain extracellular amyloid beta (Aβ) plaques and intracellular neurofibrillary tangles (NFTs), made by aggregates of hyper-phosphorylated tau. Besides memory and cognitive dysfunctions, behavioral and psychological symptoms of dementia (BPSD) are emerging as key clinical manifestations of AD. The severity of BPSD is shared in FAD and SAD, although their onset differs between the two groups, with a prevalence of BPSD higher in FAD compared to SAD [7]. In an attempt to clarify the pathogenic mechanisms leading to the development of AD, FAD-linked mutations have been exploited for the development of animal models that are useful for preclinical studies [8].

Over time, several hypotheses have been proposed to explain the pathogenesis of AD. The first and the most studied ones focus on the accumulation and deposition of Aβ plaques (amyloid cascade hypothesis [9]) and NFTs (tau hypothesis [10]). Despite immeasurable investigations performed in the last few decades, there are still no efficient disease-modifying treatments for AD [11]. Indeed, many clinical trials targeting Aβ and tau have failed, and the disease still lacks a cure [12]. More recently, other signaling pathways have also been investigated as pathogenic in AD, leading to the formulation of the infectious hypothesis, the inflammation hypothesis, the cholinergic hypothesis, the glutamatergic hypothesis, and many others (summarized here [13,14]). Various mitochondrial defects, such as decreased bioenergetics and ATP synthesis, increased ROS (reactive oxygen species) production, and altered mitochondrial transport and dynamics, have been reported to contribute to synaptic dysfunction and neuronal cell death in both SAD and FAD [15], leading to the formulation of the mitochondrial cascade hypothesis [16].

An increasing body of evidence points out the early and crucial role of cellular Ca^2+^ handling dysregulation in AD pathogenesis [17]. Interestingly, Ca^2+^ is a key regulator of several mitochondrial functions, such as ATP production, and brain cells rely mostly on OXPHOS to match their energy demands. Moreover, mitochondrial Ca^2+^ alterations may affect the functionality of other brain cells involved in memory formation and consolidation. Indeed, a growing number of studies have revealed that memory formation is not only a matter of neuronal interactions, but it also crucially involves glial cells [18]. As a result, the classical synaptic framework, consisting only of pre- and post-synaptic compartments, has been gradually expanded to include astrocytes [19] and microglia [20]. The involvement of these cell types in AD pathogenesis has also been confirmed by genome-wide association studies (GWAS). These studies describe SAD-associated genetic polymorphisms in genes involved in microglia, astrocytes and neuronal functions [21,22,23,24], as well as genes encoding mitochondria complexes or proteins involved in energy metabolism [25,26,27,28].

Thus, starting from the recent concept of quad-partite synapse and its importance in the determination of brain functions, we will here summarize the evidence of mitochondrial Ca^2+^ alterations and their consequences on cell bioenergetics in AD, focusing on neurons, astrocytes and microglia. Our overview will span different experimental disease models, focusing in particular on in vivo studies, but including also those based on Induced Pluripotent Stem Cells (iPSCs) that, by filling the gap due to the large evolutionary distance between mice and humans [29], represent a novel and promising tool to model human neurodegenerative diseases [30].

## 2. The Physiology of Brain Mitochondria: Ca^2+^ and Bioenergetics

**Figure 1:**
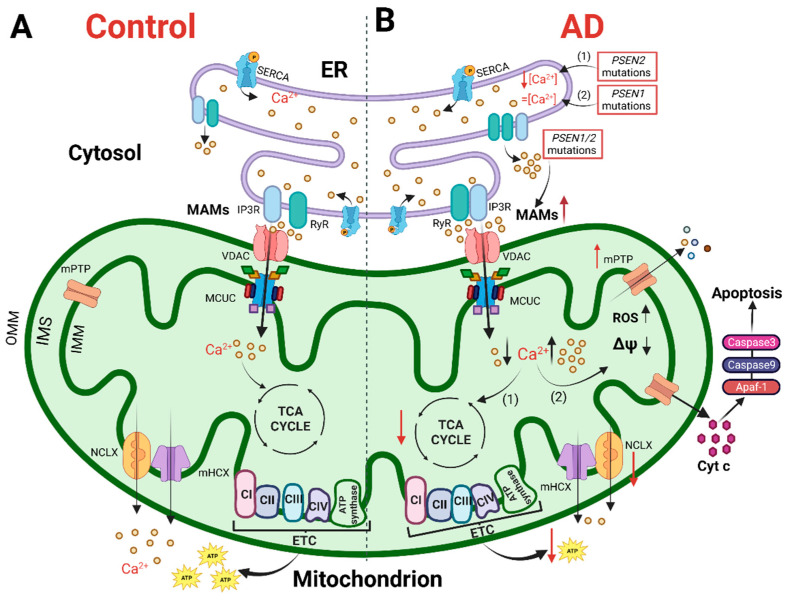
The cartoon shows mitochondrial Ca^2+^ handling and Ca^2+^-dependent bioenergetic functions in both physiological conditions (left) and AD (right). (**A**) In physiological conditions, the Ca^2+^ cross-talk between ER and mitochondria at MAMs, mediated by Inositol 1,4,5-trisphosphate receptors (IP3R) and ryanodine receptors (RyR) on the ER and VDAC and mitochondrial Ca^2+^ uniporter (MCU) complex (MCUC) on mitochondria, regulates mitochondrial Ca^2+^ entry, sustaining the TCA cycle and ATP synthesis. (**B**) In AD, various Ca^2+^ alterations have been reported. (1) Mutations in *PSEN2* cause a reduction in the ER Ca^2+^ content, whereas (2) mutations in *PSEN1* do not alter the ER Ca^2+^ content. Mutations in *PSEN1/2*, by increasing the open probability of ER Ca^2+^ releasing channels, cause an excessive release of Ca^2+^ from this store. Both a mitochondrial Ca^2+^ overload and blunted mitochondrial Ca^2+^ uptake ability have been described. The first stimulates a sustained production of ROS, the opening of mitochondrial permeability transition pore (mPTP) and the release of cytochrome c activating the apoptotic pathway. The second causes a decrease in ATP production, leading to a bioenergetic crisis. This original figure was created by the authors using “BioRender.com” (https://biorender.com, accessed on 7 November 2022).

Mitochondria are dynamic organelles able to change size, shape and position in a few seconds. They can move along the cytoskeleton to reach specific subcellular areas, or they undergo fission and fusion to constantly remodel their network and match local energy needs [31]. The organelles are equipped with a double membrane, the internal (IMM) and outer (OMM) mitochondrial membranes, which define the intermembrane space (IMS) and the mitochondrial matrix. The latter presents Ca^2+^ buffering capacity and participates in several Ca^2+^-mediated signaling pathways. The IMM is impermeable, even to small molecules, and hosts the electron transport chain (ETC) proteins devoted to OXPHOS. To maximize ATP synthesis, the IMM has many in-folding processes that span the mitochondrial matrix, where tricarboxylic acid (TCA, also called the Krebs cycle) occurs. The OMM is permeable to molecules up to 5 kDa because of the presence of the porin VDAC (Voltage-Dependent Anion Channel) [32]. This means that the IMS small molecule composition is similar to that of the cytosol, whereas molecules with a molecular weight > 5 kDa need specific transporters. The OMM is also the membrane involved in interactions with other organelles. Among them, the ER is one of the most important and its domains closely interacting with the OMM are called Mitochondria-Associated Membranes (MAMs). MAMs regulate numerous cellular processes, such as ER-mitochondria Ca^2+^ shuttling, lipid synthesis, inflammatory response, autophagy and apoptosis. These highly specialized subcellular domains are emerging as powerful signaling platforms and have been the subject of extensive studies in recent years [33]. Of note, these domains of close apposition between the ER and mitochondria result in alterations in several neurodegenerative diseases, including AD (see below and Figure 1). Although the molecular identity of proteins involved in organelle tethering is still elusive, a cell-specific molecular composition of MAMs is emerging. Recently, a neuronal-specific protein, PDZD8, has been shown to be essential for maintaining ER-mitochondria juxtaposition and neuronal Ca^2+^ homeostasis [34]. Moreover, Ooi et al. showed that in stress-induced hypertension rats, activation of the Sigma-1 receptor, a chaperone that localizes at MAMs [35], reduces microglia M1 polarization and neuroinflammation through MAM and mitochondrial activity modulation [36]. In astrocytes, MAMs are enriched at the endfeet, i.e., in the astrocyte processes in close contact with vessels [37], but no astrocytic-specific MAM proteins are known nowadays.

### 2.1. Intracellular Ca^2+^ Handling

One of the key molecules that heavily affect mitochondrial and cellular functions is Ca^2+^. The cation serves as ubiquitous intracellular messenger whose signaling is crucial for almost all aspects of cell life, such as differentiation, proliferation, growth and death [38]. Ca^2+^ is fundamental particularly for brain cells. In neurons, this ion participates in the propagation of depolarizing signals, in tuning synaptic activity, in spine and synaptic plasticity (long-term depression, LTD, or long-term potentiation, LTP) and in controlling gene transcription [39]. In astrocytes, Ca^2+^ regulates the release of gliotransmitters, such as ATP, amino acids (e.g., glutamate and D-serine) and cytokines in response to neuronal activity, thus modulating neuronal synchronization and firing. Indeed, astrocytes are tightly associated with pre- and post- synaptic elements, therefore Ca^2+^ is a key regulator of the neuron-astrocyte axis (reviewed in [40]). Finally, microglia are well known as immune sentinels of the brain, and many of their effector functions, such as motility, polarization, morphological changes and release of inflammatory mediators, are controlled by Ca^2+^ (reviewed in [41]). Thus, it is not surprising that an impaired ability of neurons and glial cells in maintaining Ca^2+^ homeostasis has also been observed in AD.

The study of brain Ca^2+^ handling is complex because neuronal/glial cells have many types of membrane-localized Ca^2+^ channels, which are activated by changes in membrane potential and a variety of ligands. Moreover, Ca^2+^ signaling literally involves many organelles to compartmentalize Ca^2+^ dynamic processes. In resting condition, intracellular Ca^2+^ concentration ([Ca^2+^]) is maintained at ~100 nM, much lower compared to that of the extracellular space, which is 1.2–2 mM. The [Ca^2+^] gradient is ensured by a complex cell-specific molecular Ca^2+^ toolkit constituted by channels, pumps and exchangers present both in the plasma membrane (PM) and in membranes of intracellular Ca^2+^ stores (mainly the ER), as well as by buffering systems [42].

In neurons, Voltage-Operated Ca^2+^ Channels (VOCCs), Receptor-Operated Ca^2+^ Channels (ROCCs), and Transient Receptor Potential (TRP) channels are responsible for Ca^2+^ entry across the PM. ROCCs can be ionotropic or metabotropic, with the latter triggering the release of Ca^2+^ from intracellular stores [39].

Astrocytes are unexcitable cells that respond to different types of stimuli through intracellular Ca^2+^ elevations mediated mainly by metabotropic or ionotropic receptors. This activity modulates both neurovascular coupling and synaptic transmission (for an extensive review, see [43]). A relevant PM ion channel in astrocytes is Transient receptor potential ankyrin 1 (TRPA1), for which a role in establishing astrocytic basal spontaneous Ca^2+^ activity has been reported [44].

A key role for Ca^2+^ signaling is also emerging in microglia functionality. Indeed, in these cells several Ca^2+^-linked receptors, activated by different pathophysiological signals released by surrounding neurons and astrocytes, such as purinergic, glutamatergic, serotonergic metabotropic receptors, but also ionotropic purinergic receptors and TRP channels, have been described (for an extensive review see [41]). Interestingly, elevation in [Ca^2+^] has been linked to executive activities of microglia, such as the production of pro- and anti-inflammatory cytokines, nitric oxide or trophic factors [45]. Major players in tuning microglia-neuron junctions are purinergic receptors, in particular P2Y12 receptors, whose activation/inhibition strongly affects this intercellular communication, indicating that P2Y12 can be a molecular determinant of microglia safeguard action of neuronal activities [46]. Generally, intracellular Ca^2+^ transients in astrocytes and microglia are mainly due to the release of Ca^2+^ from the ER [41,47].

IP3R1/2/3 and RyR (RyR3—the brain-specific isoform, although RyR1/2 have also been observed in brain tissues) are the two main types of intracellular Ca^2+^-releasing channel from the ER, obeying the bell-curve shaped Ca^2+^ release kinetics and amplifying cytosolic Ca^2+^ signals [48]. In addition to the activity of these main Ca^2+^ releasing channels, ER [Ca^2+^] is tightly controlled by several processes: (1) the activity of Sarco-Endoplasmic Reticulum ATPase (SERCA), which pumps back Ca^2+^, when released, from the cytosol to the ER lumen at the expenses of ATP hydrolysis [49]; (2) activation of Store-Operated Ca^2+^ Entry (SOCE), which refills the ER of Ca^2+^ when depleted, via the opening of the Orai1/2/3 PM Ca^2+^ channels by the interaction with the ER-located Ca^2+^ sensor, STromal Interaction Molecule (STIM) [50]; (3) “TMCO1 (Transmembrane And Coiled-Coil Domains 1)-dependent release”, a Ca^2+^-selective ion channel that undergoes a reversible homo-tetramerization in response to ER Ca^2+^ overload, and a disassembly upon Ca^2+^ depletion [51].

Upon cell stimulation, elevated [Ca^2+^] within the cell needs to quickly return to resting baseline levels to prevent cytosolic Ca^2+^ overload and cell death. SERCA and PM Ca^2+^ ATPase (PMCA, [52]) are the pumps responsible for this process, contributing to lower cytosolic [Ca^2+^] by pumping it back into the ER lumen or in the extracellular space, respectively, while consuming ATP. Besides, the Na^+^/Ca^2+^ exchanger (NCX) at the PM also aids in restoring baseline cytosolic Ca^2+^ levels. PMCA displays a higher Ca^2+^ affinity but lower capacity for Ca^2+^ transport, while the NCX shows the opposite features [42]. Despite both Ca^2+^ efflux mechanisms are involved in neuronal and astrocyte cytosolic Ca^2+^ handling, the role of PMCA in microglia is still elusive [41]. Finally, cytosolic Ca^2+^-binding proteins, like calmodulin (CaM), calbindin D-28 (CB-D28K), calretinin (CR) and parvalbumin (PV) [53,54] contribute in tuning the spatiotemporal complexity of the intracellular Ca^2+^ signal. Some of these proteins are expressed/enriched in specific cell types. For example, neuron-specific Ca^2+^-binding proteins crucial for the control of neuronal activity and the preservation of neuronal health are recoverin, neurocalcin and guanylate cyclase activating protein (GCAP) 1–3 [54]. Within the ER, the major contributors to the maintenance of its huge Ca^2+^ reservoir are ER-specific Ca^2+^ binding proteins, such as calreticulin (CRT) and calnexin (CNX) [55].

### 2.2. Mitochondrial Ca^2+^ Handling

In the complex scenario of cellular Ca^2+^ homeostasis, mitochondria have a role as the most important organelle able to modulate cytosolic Ca^2+^ rises, thanks to their ability to transiently take up Ca^2+^ [56]. This skill depends on the negative mitochondrial membrane potential (−180 mV) and the presence of MCUC. The latter is a high capacity, low affinity (K_d_∼15 μM) Ca^2+^ channel located in the IMM [57,58]. Importantly, depending on the placement of these organelles with respect to the source of Ca^2+^, i.e., intracellular Ca^2+^ releasing channels or PM-located Ca^2+^ channels, the amplitude and kinetics of mitochondrial Ca^2+^ uptake varies. Indeed, the surge in cytosolic [Ca^2+^] upon stimulation is generally between 1–3 μM, which cannot efficiently trigger the opening of the MCU. However, near the mouth of Ca^2+^ permeable channels and receptors, such as PM channels, IP3Rs and RyRs, microdomains of high [Ca^2+^] occur, enabling the rapid opening of the MCUC [59] (Figure 1A). Recent data have revealed a very complex molecular composition of the MCUC, where the pore-forming component MCU is controlled by numerous regulatory units: (1) MICU1, the gatekeeper of MCU; (2) MICU2, an inhibitor of MCU, endowed as MICU1, with an EF-hand domain, thus mediating the Ca^2+^-dependent regulation of MCU; (3) MCUb, the dominant negative component of the channel; (4) EMRE, a protein crucial in MCUC assembly [60]. Different cell type-specific isoforms of these molecules exist and MICU3, an MCU facilitator, is mainly expressed in the brain. MICU3 activates MCU at lower Ca^2+^ levels leading to an increased axonal mitochondria ability to take up Ca^2+^ and sustain ATP synthesis and synaptic functionality [61,62].

Once taken up by mitochondria, Ca^2+^ is released back to the cytosol due to the activity of Na^+^/Ca^2+^ (NCLX) [63] and H^+^/Ca^2+^ (mHCX) exchangers, whose molecular identity has recently been identified [64]. NCLX (NCX1–3 in brain cells), mostly expressed in excitable cells, is a low affinity, high capacity transporter that extrudes 1 Ca^2+^ molecule from mitochondria by using the electrochemical gradient of 3 molecules of Na^+^ (or Li^+^) [65].

Many in vitro studies have reported the ability of brain mitochondria to take up Ca^2+^, and in neurons, this ability seems to be required for both ATP production (Figure 1A) and buffering of cytosolic Ca^2+^. The emerging picture is that changes in MCUC activity are required to tune the neuronal firing rate [66] and synchronization of neuronal spiking [67]. The reliability of neuronal activity on mitochondrial Ca^2+^ uptake can differ among brain regions as a function of their distinct metabolic profiles or different MCUC compositions. Only recently has mitochondrial Ca^2+^ uptake been investigated in vivo in the central nervous system (CNS), where the coupling of cytosolic and mitochondrial Ca^2+^ occurs in a non-deterministic manner via CaMKII signaling. Of note, this coupling is more efficient during task performance, further matching the notion that mitochondrial Ca^2+^, by regulating mitochondrial bioenergetics, satisfies neuronal energy needs and contributes to neuronal information processing [68].

The biophysical properties that govern mitochondrial Ca^2+^ uptake and efflux, as well as the molecular Ca^2+^ toolkit, are conserved in glial cells [69], where, instead, the physiological (and pathological) relevance of mitochondrial Ca^2+^ buffering is not completely characterized. Mitochondria Ca^2+^ buffering has been reported to strongly contribute to the control of astrocytic global Ca^2+^ signals. Accordingly, blocking mitochondrial Ca^2+^ uptake in astrocytes slows the decay rate of Ca^2+^ transients and increases the propagation of Ca^2+^ signals [70].

To the best of our knowledge, no data about the connection between mitochondrial Ca^2+^ handling and microglia functionalities are available^,^ although a crucial role of mitochondrial Ca^2+^ handling in inflammation and cell polarization has been demonstrated in cell lines or in macrophages [71,72].

### 2.3. Mitochondria, Ca^2+^ Hotspots and Ca^2+^ Microdomains

Ca^2+^ hotspots are small cellular areas of high [Ca^2+^] typically generated at sites where, upon stimulation, Ca^2+^ either enters the cell at the PM or is released from intracellular stores. Their formation is crucial for cell physiology and for mitochondrial Ca^2+^ homeostasis. Indeed, efficient mitochondrial Ca^2+^ uptake requires Ca^2+^ hotspot formation, and the buffer of Ca^2+^ by mitochondria at these sites is required for the spatiotemporal modulation of intracellular Ca^2+^ dynamics [73]. In neurons, Ca^2+^ hotspots occur in the different decoding functional units where mitochondria concentrate, providing the ATP required to accomplish neuronal function. Of note, in sensory neurons, 40% of Ca^2+^ clearance is mediated by mitochondria [74], and the organelle are able to take up Ca^2+^ in both neuronal soma and dendrites, where Ca^2+^ hotspots occur. In particular, Ca^2+^ elevation in dendritic spines is involved in neuronal plasticity, whereas Ca^2+^ hotspots at synaptic terminals control neurotransmitter release [75,76,77,78]. Recently, taking advantage of a MCU haplo-insufficient mouse model, it has been shown that the reduced mitochondrial Ca^2+^ buffering capacity (due to the reduction in MCU protein levels) impacts the presynaptic Ca^2+^ clearance, increasing the release probability of neurotransmitters, despite reduced ATP production [79].

The functional role of these Ca^2+^ hotspots remains unexplored in glial cells. Only recently, Marsicano’s laboratory described the role of mitochondrial-associated type-1 cannabinoid (mtCB_1_) receptors activation in mediating the IP3R-dependent Ca^2+^ transfer from ER to mitochondria. The molecules involved in this transfer, besides IP3R, are AKT, MICU1 and MCU. The mtCB_1_-mediated Ca^2+^ transient is involved in hippocampal lateral synaptic potentiation (LSP), a mechanism through which astrocytes integrate the activity of distant synapses [80].

Ca^2+^ hotspot formation is a ubiquitous phenomenon, and these domains of very high [Ca^2+^] are essential for mitochondrial Ca^2+^ uptake and ATP synthesis in all brain cells. On the other hand, mitochondria are also reported to play a role in determining the formation of other Ca^2+^ microdomains spatially restricted within the cell and occurring spontaneously in glial cells, especially in astrocytes. These localized Ca^2+^ events were first described as Ca^2+^ microdomains in the Bergmann glia processes [81]. In astrocytes, spatially restricted Ca^2+^ microdomains occur spontaneously and are localized in the fine protrusions close to the synapses, thus providing an ideal position to act on synaptic transmission. For this reason, spontaneous Ca^2+^ microdomains are becoming the focus of several recent studies in an attempt to clarify both the mechanisms at the basis of this type of Ca^2+^ activity and the functional significance (for an extensive review on this, see [47,82,83]). To the best of our knowledge, no experimental studies have been performed to assess the actual [Ca^2+^] in astrocyte Ca^2+^ microdomains because of technical limitations. Notably, a computational study revealed that these domains in astrocyte present a high [Ca^2+^], in the order of 1–2 μM well above the overall basal [Ca^2+^] ranging around 60–80 nM [84]. Importantly, different works have shown the presence of mitochondria in astrocyte fine protrusions in situ and in vivo [70,85,86]. Indeed, cytosolic Ca^2+^ microdomains in astrocytes frequently co-localize with mitochondria in hippocampal organotypic slices, suggesting a possible role of mitochondrial Ca^2+^ handling in sustaining this phenomenon. On the same line, by elegant in vivo 2-photon imaging experiments, Agarwal and co-workers confirmed the co-localization of a significant portion of Ca^2+^ microdomains with mitochondria (around 85%) in astrocyte and showed that part of the spontaneous microdomain generation could be blocked by inhibiting the mPTP [85], strongly indicating that spontaneous Ca^2+^ microdomains are partially due to Ca^2+^ efflux from mitochondria.

Microglial cells exhibit less spontaneous and very infrequent Ca^2+^ transients in basal conditions. However, microdomains of Ca^2+^, despite being rare, are also reported in microglial processes in response to variations in neuronal activity. In contrast, frequent microglial somatic Ca^2+^ transients have been linked to longitudinal epilepsy development [87].

### 2.4. Mitochondrial Ca^2+^ Signaling and Bioenergetics: The Energy Match

The most abundant energy molecule within the cell is ATP, which might be, depending on the cell type, a product or a byproduct of various metabolic pathways, such as glycolysis, the TCA cycle, the fatty acid β-oxidation pathway, the pentose phosphate pathway, and the urea cycle. Excitable cells mainly rely on OXPHOS to produce ATP [88].

Mitochondria are endowed with a unique ultrastructure that lets them produce ATP. Chemiosmotic theory states that ATP is synthesized from glucose by processing through glycolysis and the TCA cycle to two reducing equivalents, NADH and FADH_2_ that further transfer electrons to ETC via redox reactions. Meanwhile, transporting electrons, the ETC complexes actively pumping protons from the matrix to the IMS generate an exergonic electrochemical gradient exploited by the ATP-synthase to synthesize ATP from ADP and phosphate [89]. IMS and mitochondrial matrix Ca^2+^ regulates mitochondrial metabolism (Figure 1A). Generally, an increase in the mitochondrial Ca^2+^ is paralleled by increased respiration, NADH generation and ATP production. In the 70s, it was discovered that pyruvate dehydrogenase (PDH), an enzyme that converts pyruvate into acetyl-CoA, is regulated by Ca^2+^, and that the activity of the TCA cycle enzymes isocitrate dehydrogenase (IDH) and oxoglutarate dehydrogenase (OGDH) is directly modulated by Ca^2+^ binding. These three proteins are located in the matrix, and thus, they respond to changes in [Ca^2+^] in this sub-compartment. Additionally, several mitochondrial carriers located in the IMM are regulated by Ca^2+^. This is the case of flavin adenin nucleotide-glycerol phosphate dehydrogenase (FAD-GPDH), Aralar and the ATP-Mg/P_i_ carrier. FAD-GPDH transfers reducing equivalents from cytosolic NADH to the ETC, sustaining mitochondrial respiration; Aralar is a glutamate/aspartate antiporter, involved in glutamate-dependent mitochondrial respiration, and ATP-Mg/P_i_ carrier exchanges ATP-Mg (or ADP-Mg) and phosphate (P_i_), controlling the content of mitochondrial adenine nucleotides. Furthermore, Ca^2+^ is emerging as a key regulator of nucleotide, metabolite and cofactor shuttling, mediated by L-CAMCs (Long Ca^2+^-dependent mitochondrial carriers for aspartate/glutamate) and S-CAMCs (Short Ca^2+^-dependent mitochondrial carriers of ATP-Mg/P_i_), both able to sense changes in IMS [Ca^2+^]. Despite debate, some works have reported a direct effect of Ca^2+^ on ETC complexes and ATP-synthase. In line with all these findings, the constitutive, low level ER-mitochondria Ca^2+^ transfer has been reported to be pivotal in maintaining cell ATP production (reviewed in [88,90]).

To maintain their activity, neurons rely heavily on mitochondrial ATP synthesis. It has been demonstrated that Ca^2+^ entrance into neuronal mitochondria regulates energy production in an activity-dependent manner [91]. Similarly, synaptic activity requires and induces ATP synthesis [92], with mitochondrial Ca^2+^ oscillations linked to synaptic activity, creating precise requirement-synthesis matching. The coupling of mitochondrial ATP generation with bioenergetic demands in neurons is still poorly understood in its molecular mechanisms. Recently, Zampese et al. elegantly showed that in substantia nigra dopaminergic neurons spike-activated Ca^2+^ entry through Ca_v_1 channels triggered Ca^2+^ release from the ER, which in turn stimulated mitochondrial OXPHOS through the MCUC and the malate-aspartate shuttle. Disruption of this mechanism impaired the ability of dopaminergic neurons to sustain both spike activity and bioenergetic demands, leading to oxidative stress and neuronal damage [93]. Interestingly, MCUC activation, promoting ETC activity and the reduction of NAD^+^ (Nicotinamide adenine dinucleotide) to NADH, has also been reported to be required for pyramidal neuron firing [66]. Another recent paper, instead, showed that the Aralar-malate-aspartate shuttle during neuronal activation boosts glycolysis, pyruvate transport and OXPHOS in an MCUC-independent manner, but with the involvement of the Ca^2+^-dependent regulation of Aralar itself [94].

Different from neurons, ATP production in astrocytes mostly depends on aerobic glycolysis [95], although these cells also partially rely on mitochondria for ATP production. Indeed, OXPHOS in astrocytes accounts for 30% of the whole O_2_ consumption in the brain [96]. One of the main functions of astrocytes in the CNS is to take up glutamate from the extracellular space to avoid neuronal excitotoxicity. Glutamate uptake is mediated by the astrocyte-specific glutamate transporter GLT-1 (Glutamate transporter-1, also known as Excitatory Amino Acid Transporter-2, EAAT2) through a Na^+^ dependent co-transport [97]. Astrocytes consume 20% of their ATP to sustain the activity of the Na^+^/K^+^ ATPase, which is necessary to maintain the Na^+^ gradient across the PM [98]. Of note, GLT-1 and the Na^+^/K^+^ ATPase co-localize with mitochondria and glycolytic enzymes [99,100].

In microglia, the role of mitochondrial Ca^2+-^dependent bioenergetics in determining their functionalities is more complicated. Transcriptomic data indicate that microglia express the genes required for both glycolysis and oxidative metabolism [101]. Growing results suggest that microglia typically experience a unique metabolic shift during their activation [102], i.e., in their homeostatic state, they rely mainly on OXPHOS, whereas pro-inflammatory stimuli shift microglia metabolism toward glycolysis [103,104,105]. However, other studies indicate that microglia exposed to anti-inflammatory stimuli [IL (Interleukin)-4, IL-13, IL-10, TGF (transforming growth factor) -β and glucocorticoids] exhibit oxygen consumption rate (OCR), basal respiration and ATP generation quite similar to those of homeostatic, not treated, microglia [106,107]. Furthermore, the microglial metabolic state depends on the type of activator (pathogen-associated molecular patterns, PAMPs, or damage-associated molecular patterns, DAMPs). In primary cultured microglia, LPS, a PAMP, greatly increased glycolysis while suppressing OXPHOS. On the other hand, extracellular ATP, a well-recognized DAMP, increases both glycolysis and OXPHOS. Thus, it seems that different microglia states and different kinds of activators specifically modulate their metabolic state [108].

## 3. Mitochondria in Alzheimer’s Disease: Ca^2+^ Signaling and Bioenergetics

More than 30 years ago, Khachaturian first described a Ca^2+^ homeostasis alteration in different AD cells [109,110]. From this original observation, the so-called “Ca^2+^ cascade hypothesis for AD” has been proposed [111], in which neurons from aged and diseased brains experience cytosolic Ca^2+^ overload upon depolarization [112]. Furthermore, mitochondrial Ca^2+^ alterations, as well as dysfunctional mitochondria, have been reported in various models of AD, including postmortem patient-derived samples, leading to the formulation of the “mitochondrial cascade hypothesis for AD” [113]. Based on this latter, excessive mitochondrial Ca^2+^ uptake triggers an increase in ROS production, ATP synthesis inhibition, mPTP opening, release of cytochrome c, activation of caspases and apoptosis (revised in [90], Figure 1B). However, contrasting data have also been produced, and this scheme is not completely accepted. Nowadays, there are still few works investigating the role of mitochondria in the pathogenesis of AD in physiologically relevant experimental samples, i.e., ex vivo and in vivo. Furthermore, one of the main issues to be addressed for both the Ca^2+^ and mitochondrial cascade hypotheses is to define whether Ca^2+^ or mitochondrial dysfunctions have a primary and causative role in AD pathogenesis. Indeed, to gain insight into new potential therapeutic targets, it is crucial to determine whether one of these defects is sufficient *per se* to cause AD. Since the mitochondrial ability to take up Ca^2+^ strongly depends on cytosolic Ca^2+^ dynamics, both parameters must be assessed in the same model. Indeed, many global Ca^2+^ alterations have been reported so far in various AD models.

### 3.1. Intracellular Ca^2+^ Signaling Alterations in AD

Various studies have described a synergistic and bidirectional action of Aβ and Ca^2+^, where Aβ accumulation triggers Ca^2+^ signaling alterations and the latter promotes Aβ production. In vitro, ex vivo and in vivo studies in neurons report that Aβ oligomers increase cytosolic Ca^2+^ levels by acting on PM-located channels, such as metabotropic glutamate receptors (mGluRs), Orai, N-methyl-D-aspartate ionotropic glutamate receptors (NMDARs), nicotinic acetylcholine receptors (7-nAChRs) and VOCCs. However, Aβ has been additionally shown to increase neuronal cytosolic [Ca^2+^] by inhibiting PMCA and NCX (reviewed in [114,115]).

Multiple data have been collected in the last decades linking *PSEN* mutations to Ca^2+^ alterations and cell death. Presenilin 1 and 2 (PS1 and PS2) form the catalytic core of γ-secretase complex, an enzyme mainly located in the ER membranes and participating in APP cleavage [116]. However, PS functions are not limited to their γ-secretase activity, and the two proteins alone can instead modulate different cell processes, included Ca^2+^ homeostasis. In the presence of mutated PS1 or PS2, different Ca^2+^ pathways are altered. Initially, data obtained mainly in FAD-PS1-expressing samples showed an increase in the ER Ca^2+^ content, thus causing excessive Ca^2+^ release in the cytosol upon cell stimulation. These data led to the formulation of the Ca^2+^ overload hypothesis for AD, in which a higher ER [Ca^2+^] causes both altered APP processing, with an accumulation of Aβ toxic peptides, and deregulated kinase activity, leading to tau hyper-phosphorylation and eventually to a Ca^2+^-dependent neuronal death [111]. However, recent evidence (obtained in FAD-PS2-expressing models) disproves the PS-linked ER Ca^2+^ overload, leading to a revision of the pathogenic hypothesis (revised in [117]). In addition, an impairment in SOCE has been reported in many FAD-PS cellular models, including patient-derived samples [118,119,120]. Nowadays, the most accepted view is that, in AD models, there is an altered Ca^2+^ handling due to an excessive release of the cation from the ER (Figure 1B), an inhibited ER Ca^2+^ re-uptake and a decreased ER Ca^2+^ refilling (due to a reduced SOCE), independently from the ER Ca^2+^ content [117]. On this line, recent studies on human iPSC-derived basal forebrain cholinergic neurons (BFCNs)—among the most vulnerable cells to early degeneration in AD—from FAD patients with the *PSEN2*-N141I mutation showed a reduction of both spike frequency and spike amplitude [121] and a reduced insulin-triggered cytosolic Ca^2+^ transient [122].

Alterations of cytosolic Ca^2+^ signaling also occur in astrocytes of different AD models. Hippocampal brain slices from 2-week-old PS2APP mice (carrying mutation in both *PSEN2* and *APP* genes) showed a reduced response of astrocytes to mGluR stimulation, well before the onset of Aβ plaque deposition [123]. In APP/PS1-based models, instead, cortical astrocytes are characterized by spontaneous hyperactivity mediated by P2Y1R only after Aβ plaque deposition [124,125]. In the same mouse model, however, astrocyte Ca^2+^ hypo-responsiveness to sensory stimuli has also been reported [126]. Conversely, APP^NL-F^ mice showed decreased astrocyte Ca^2+^ activity correlated with neuronal hyperactivity in the early phase of the disease. Interestingly, the rescue of astrocyte Ca^2+^ activity through chemogenetic tools is also able to recover neuronal deficits [127]. An attenuated cytosolic Ca^2+^ activity in response to locomotion has also been reported in the astrocyte of tg-ArcSwe mice with overt Aβ plaque deposition [128]. Finally, an increased Ca^2+^ release from the ER was observed in *PSEN1*Δ9 iPSCs-derived astrocytes [129]. Altogether, these studies point to different types of astrocytic Ca^2+^ dysfunctions in AD mice, at least in the cytosol, and most of these alterations occur after Aβ plaque deposition.

Microglia involvement in AD has been widely explored, both in vivo and in postmortem samples of AD patients. In both models, activated microglia surrounded Aβ plaques [130]. Microglia isolated from postmortem human AD brains show higher basal intracellular [Ca^2+^], compared to controls [131]. Multiple in vitro data suggest that stimulation of microglial cells, for example, with lipopolysaccharide (LPS) or Aβ, causes a prolonged rise in intracellular [Ca^2+^]. This cytosolic Ca^2+^ overload has been attributed to purinergic P2X7 receptor activation, whose expression increases in AD microglia [132]. In addition, Calmh2 (Ca^2+^ homeostasis modulator ion channel) has been reported to be overexpressed in the activated microglia of 5xFAD mice and its knockdown mitigates neuroinflammation [133,134]. The activated microglia in AD mice and humans display a reduction in the cytosolic Ca^2+^ transients evoked by ATP, UTP and complement factor C5a [131]. Finally, analysis of Ca^2+^ signaling in human iPSC-derived microglia with genetic deletion of TREM2 (Triggering Receptor Expressed on Myeloid cells 2, a molecule whose rare loss-of-function variants associate with increased risk of AD) revealed higher cytosolic Ca^2+^ transients in response to ADP, possibly due to a greater release of Ca^2+^ from the ER and an increased SOCE [135]. Interestingly, an in vivo study performed in an APP/PS1 model of AD shows that, in the vicinity of Aβ plaques, microglial cells display frequent somatic Ca^2+^ rises linked to purinergic receptor hyperactivity [136].

Overall, an increase in cytosolic Ca^2+^ rises has been reported in different PS1- and APP-based models, whereas PS2-based models mainly displayed a decreased Ca^2+^ transient upon cell stimulation due to the partial depletion of ER [Ca^2+^] caused by the mutated protein.

### 3.2. Mitochondrial Ca^2+^ Signaling Alterations in AD

**Figure 2:**
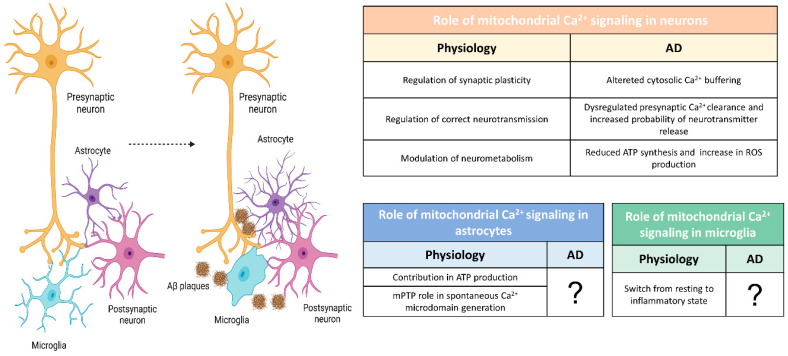
(**Left**), schematic representation of the cellular elements involved in the formation of the quad-partite synapse. Transition from healthy to AD condition with the presence of Aβ plaques and the morphological changes in astrocytes and microglia. (**Right**), tables reporting the roles of mitochondrial Ca^2+^ signaling in neurons, astrocytes and microglia and the reported dysfunctions in different AD samples. This original figure was created by the authors using “BioRender.com” (https://biorender.com, accessed on 7 November 2022).

The defects in Ca^2+^ influx across the PM or its release from the ER, described above in different AD samples, can impact mitochondrial Ca^2+^ handling. However, mitochondrial Ca^2+^ can also be altered per se and this alteration could affect the global Ca^2+^ signaling. Generally, the accumulation of Ca^2+^ into the mitochondrial matrix can trigger cell death by the release of cytochrome c and the activation of caspase 9 and 3 (Figure 1B) [88,137]. However, a blunted mitochondrial Ca^2+^ signal can also be detrimental for both the consequent decreased ATP production and the altered cytosolic Ca^2+^ buffering (Figure 1B and Figure 2). For example, it has been recently shown that in neurons downregulated for either MCU or mitochondrial pyruvate carriers 1 (MPC1), as a consequence of blunted mitochondrial Ca^2+^ uptake, the cytosolic Ca^2+^ level was significantly higher compared to the control, contributing to neuronal hyperexcitability [66,138].

Summarizing several works performed in different AD-related models, two opposite mitochondrial phenotypes (Figure 1B) have been reported so far: (1) a mitochondrial Ca^2+^ overload, mainly in cells treated with Aβ oligomers and in FAD-PS1-based mouse models; (2) a blunted mitochondrial Ca^2+^ uptake, mainly in FAD-PS2-based mouse models (Figure 1B). In vitro data report that Aβ oligomers cause mitochondrial Ca^2+^ overload in neurons [139,140], similarly to what has been found in the cytosol (see above). One of the molecular mechanisms proposed to explain Aβ-mediated mitochondrial Ca^2+^ overload contemplates Aβ translocation in the mitochondria, where it can favor the opening of mPTP (Figure 1B) [141]. mPTP is a high-conductance mitochondrial channel whose composition and structure are still up for discussion [142]. mPTP opens in response to mitochondrial matrix Ca^2+^ excess or when oxidative stress is present [143]. Aβ is reported to interact with cyclophilin D, a crucial component of the pore, enhancing mPTP open probability [144]. Accordingly, a reduction in cyclophilin D expression and/or treatment with cyclosporine A (which instead blocks mPTP) enhances learning and memory and protects neurons from cell death [144,145]. It has to be noted, however, that studies linking exogenous administration of Aβ oligomers with mitochondrial Ca^2+^ overload need to be validated in vivo, as the synthetic Aβ used at very high concentrations or dissolved in glutamate-enriched media. Furthermore, suggesting a direct Aβ effect on mitochondria, a topological paradox emerges. The processing of APP foresees the secretion of its Aβ products in the extracellular space or embedded in the lumen of secretory vesicles. To directly affect mitochondria, Aβ should exit the vesicles and be imported into the mitochondria. Recently, this paradox has been partially solved, showing that APP can be processed at MAMs [146], although the mechanisms through which Aβ passes several biological membranes remain elusive.

Contrasting results on mitochondrial Ca^2+^ signaling have also been published regarding AD-linked *PSEN*1 mutations, with data sustaining the mitochondrial Ca^2+^ overload hypothesis in neurons isolated from PS1-transgenic mice [147], and others reporting impaired mitochondrial Ca^2+^ uptake in cultured rat hippocampal neurons transfected with *PSEN1*-M146V [148]. Hippocampal neurons from PS2-mutant-based models, instead, show a slower return to resting mitochondrial [Ca^2+^] upon the application of a mild excitotoxic stimulus [149].

As PSs are enriched in MAMs [146,150], several works have focused on the role of these proteins, as well as of Aβ oligomers, in regulating the juxtaposition between ER and mitochondria.

Analyses of postmortem cerebellar tissues from patients with *PSEN1*-E280A revealed abnormal ER-mitochondria tethering and mitochondrial distribution, as well as severely decreased levels of Ca^2+^ channels (P/Q-type VOCCs) and IP3R1 and IP3R3 [151]. Similarly, two important MAM proteins, VAPB and PTPIP51, as well as IP3R1, have been reported to be reduced in the temporal cortex of human post-mortem AD brains at later stages of the disease (Braak stage VI). However, the VAPB-PTPIP51 ER-mitochondrial tether has also been found to be reduced in the early Braak stages (III-IV) [152].

Different AD models display an increased physical interaction between ER and mitochondrion [123,151,153,154,155,156], even before Aβ plaque deposition [123,157], although other reports indicate a decrease in ER-mitochondria contact sites in cells treated with Aβ oligomers [158], or no effect by the expression of *PSEN1* mutations [153]. Generally, it has been demonstrated that there is a positive correlation between the number of ER-mitochondria contacts and aging [159].

Only recently have in vivo studies been performed to address mitochondrial Ca^2+^ signaling deregulation in AD. Jadiya et al. showed that AD progression, in the 3xTg AD mouse model (mutated in PS1, APP and tau), is associated with the progressive loss of the mitochondrial NCLX. It is noteworthy that this loss started before Aβ plaque deposition, and neuronal overexpression of NCLX completely restored brain Aβ plaque load, tau hyperphosphorylation and cognitive decline [160]. Furthermore, Calvo-Rodriguez and colleagues show a higher basal level of mitochondrial Ca^2+^ in a similar model, only after Aβ plaque deposition, i.e., at 8–9 months of age. This phenotype has been attributed to an increase in MCU expression levels, as its lowering rescued most of the AD hallmarks [161].

To the best of our knowledge, no studies have been performed to assess mitochondrial Ca^2+^ dynamics in vivo in PS2-based mice, as well as in astrocytes or microglia in any AD model. Instead, in vitro primary cultured astrocytes lacking APP show fragmented mitochondria with altered Ca^2+^ uptake after ATP stimulation [162]. Considering the important role of Ca^2+^ and mitochondria in glial cell physiology, this aspect deserves future proper studies.

### 3.3. Mitochondrial Bioenergetics in AD

Many alterations in mitophagy, mitochondrial dynamics and ETC protein level/activity have been reported so far in many AD models (for an extensive review, see [15,163]). Here, we will discuss the bioenergetic consequences of mitochondrial Ca^2+^ deregulations in AD reported so far (Figure 2). Indeed, among the multiple functions of mitochondria, ATP production is a key Ca^2+^-regulated event.

The role of PSs in regulating mitochondrial function has been addressed in several in vitro studies. PSs, by modulating MAMs, can indeed impact mitochondrial Ca^2+^ handling and, thus, mitochondrial bioenergetics. The two mitochondrial Ca^2+^ phenotypes reported in AD models (a mitochondrial Ca^2+^ overload or a blunted mitochondrial Ca^2+^ signal; see above) can both culminate in cell death, thus contributing to neurodegeneration via two different mechanisms. In this context, the Ca^2+^ shuttling between the ER and mitochondria is of particular interest. Indeed, it has been shown that even a reduced basal shuttling of this cation between the two organelles can increase the phosphorylation of PDH, inhibiting the enzyme and slowing down the TCA cycle, reducing the availability of NADH and FADH_2_, thus impairing ETC activity. This process reduces ATP production, culminating in autophagy activation (reviewed in [88]).

However, a direct correlation between mitochondrial Ca^2+^ alteration and bioenergetic deficits is lacking in most of the studied AD models. Some of this information can be inferred by combining data obtained in different studies and performed in AD mouse models based on the same mutations. For example, mitochondrial functional analyses in the 3xTg-AD mouse model, characterized by an age-dependent reduction in NCLX expression [160], displayed decreased mitochondrial respiration and a reduction in PDH protein levels and activity in young female mice [164]. Interestingly, a mouse model of PDH deficiency specifically shows decreased fast-spiking neuron excitability, thus providing a possible link between mitochondrial defects and cell-neuronal hyperexcitability [165].

Severe mitochondrial impairments were observed in neurons in proximity of Aβ plaques of an APP/PS1 AD mouse model [166]. In a similar model, the basal mitochondrial Ca^2+^ level has been found to be increased after Aβ plaque depositions [161].

Our group recently linked mitochondrial Ca^2+^ defects induced by *PSEN2* mutations to bioenergetics. In particular, Rossi et al. showed that in different cell types expressing mutated *PSEN2,* blunted mitochondrial Ca^2+^ uptake, together with decreased mitochondrial pyruvate import, contributed to reduced ATP production (Figure 2) [167]. In the work of Rigotto et al., the impaired mitochondrial Ca^2+^ efflux, described in cultured hippocampal neurons isolated from a PS2/APP mouse model, is linked to lower maximal respiration and decreased ability to sustain the membrane potential of neuronal mitochondria [149]. Cortical neurons from the same model also show an impaired glycolytic flux that causes mitochondrial hypometabolism [168].

If the direct link between mitochondrial Ca^2+^ alterations and bioenergetics is still largely unexplored in neurons, the panorama is even more limited in glial cells, where the study of mitochondrial defects has not yet been addressed (Figure 2). In 5xFAD mice, TREM2^-/-^ microglia were characterized by a lower number of mitochondria and a lower ATP level [169]. On the same line, the treatment of TREM2 deficient mice with sodium rutin, to increase ATP production and OXPHOS, enhances the ability of microglia in clearing Aβ, while OXPHOS reduction by rotenone/antimycin A treatments affects the ability of these cells to phagocyte Aβ [170].

## 4. Mitochondria Ca^2+^ Signaling and Bioenergetics as Therapeutic Targets

Since mitochondrial Ca^2+^ signaling is pivotal in determining many brain cell functions, its therapeutic targeting can be exploited to avoid neurodegeneration and cell death. The recent discovery of the molecular components of MCUC expands the possibility to tune mitochondrial Ca^2+^ handling. Nowadays, many drugs targeting MCUC are available, but most of them are not able to cross the blood–brain barrier or they are prone to multiple side effects. Concerning mitochondrial Ca^2+^ uptake, the historical inhibitor and cell-impermeant ruthenium red have been modified, obtaining a more permeable and powerful version called Ru265 [171]. High-throughput screening analyses are unveiling novel inhibitors of MCUC, such as Mitoxantrone [172], DS16570511 [173], MCU-i4 and MCU-i11 [174]. Recently, amorolfine and benzethonium, positive and negative MCUC modulators, respectively, have been identified by high-throughput screening on a US Food and Drug Administration (FDA)-approved drug library [175].

Natural plant flavonoids have been reported to activate MCU. Kaempferol is one of the compounds reported to exert a protective effect on mitochondrial functions by activating MCU [176]. Furthermore, kaempferol has been shown to improve the metabolism-secretion coupling of β-pancreatic cells [177]. SB202190, an inhibitor of p38 mitogen-activated protein (MAP) kinase, has also been reported to reversibly stimulate mitochondrial Ca^2+^ uptake [176]. Antioxidants have been reported to positively modulate mitochondrial Ca^2+^ homeostasis, by stabilizing the cellular redox state or targeting mitochondrial ROS (reviewed in [178]).

As for mitochondrial Ca^2+^ efflux, the NCLX inhibitor CGP-37157 is widely used despite its lack of specificity [179].

Furthermore, different therapeutic possibilities are expanding over time, showing promising tools for modulating mitochondrial function (reviewed here [180]). Gene therapy can be exploited to manipulate specific genes expressed only in a cell type, targeting either neurons, astrocytes or microglia, as well as to specifically edit mitochondrial DNA (mtDNA) without altering genomic DNA. Finally, diet and exercise can enhance/modulate mitochondrial function. As an example, ketogenic diets have been reported to rescue neuronal Ca^2+^ handling dysfunction, by overcoming the mitochondria hypometabolism caused by impaired pyruvate import [138].

## 5. Conclusions

A very complex scenario is emerging on mitochondrial Ca^2+^ homeostasis in the brain, with the organelle Ca^2+^ signal not only crucial for cell metabolism and bioenergetics but also fundamental for shaping overall cytosolic Ca^2+^ handling. Very little is known about the role of mitochondrial Ca^2+^ buffering in the regulation of global Ca^2+^ signal in glial cells. Even in neurons, this aspect is overlooked. Recent works highlight a close correlation between mitochondrial Ca^2+^ buffering and neuronal activity, pointing toward neuronal subtype-specific regulation of mitochondrial Ca^2+^ signaling. In this context, mitochondrial functional analysis in AD is even more puzzling because of the difficulties of fully phenocopying AD traits and progression, the lack of systematic transversal and longitudinal studies of mitochondrial Ca^2+^ handling and related pathological consequences in physiologically relevant contexts, and the absence of correlative studies between cytosolic and mitochondrial Ca^2+^ dynamics. Despite the multiple hypothesis, nowadays, AD is considered a multifactorial pathology and mitochondria could have a pivotal role. The continuous development of indicators that enable the measurement of the dynamic changes of intracellular ligands in real time and in complex systems, together with the possibility of pharmacologically and genetically modulate mitochondrial Ca^2+^ handling and bioenergetics, constitute and will constitute a great number of tools to investigate the pure role of mitochondrial (dys) function in the pathogenesis and progression of AD.
